# Adrenal Crisis in a Delayed Diagnosis of Sheehan Syndrome

**DOI:** 10.7759/cureus.44225

**Published:** 2023-08-27

**Authors:** Patrick Powers, Kathryn Jan, Deepak Bommisetty

**Affiliations:** 1 Medical Education, Burnett School of Medicine at Texas Christian University, Fort Worth, USA; 2 Anesthesiology, University of Texas Southwestern Medical Center, Dallas, USA; 3 Internal Medicine, Methodist Dallas Medical Center, Dallas, USA

**Keywords:** sheehan syndrome, endocrinology, pituitary infarction, obstetrical hemorrhage, adrenal crisis

## Abstract

Sheehan syndrome is a well-documented endocrinological disorder that appears to be closely associated as a secondary sequela to postpartum hemorrhage. Due to pregnancy-related physiological adaptations, namely the increase in blood volume but lack of hypertrophic or hyperplastic growth within the pituitary, pregnancy increases the likelihood of infarction of the pituitary. This, coupled with other complications, such as postpartum hemorrhage, can lead to ischemia and permanent damage to the pituitary, and thus, all the downstream endocrinological pathways regulated by the pituitary. Namely, this can include, but is not limited to, adrenal crisis from improper stimulation of steroid secretion. Individuals who have been diagnosed with Sheehan syndrome require lifelong steroid supplementation for appropriate regulation of multiple systems, specifically circulatory. Without appropriate steroid supplementation exogenously, patients can rapidly decline with adverse hypotension, altered mental status, and loss of vascular tone. This case presents a case of a patient who, after extensive chart review and history taking, was found to have had a complicated pregnancy many years ago with multiple transfusions needed to stabilize her and was placed on exogenous steroid management, presenting for adrenal crisis, hypotension, and altered mental status after not taking her home steroid medication.

## Introduction

Sheehan syndrome is characterized by localized infarction of the pituitary gland, most notably as a result of post-partum hemorrhage. Maternal gestational changes and adaptations include relative hyperplasia of the pituitary gland with minimal increase in blood supply to the hypothalamohypophyseal portal system due to increased blood flow being prioritized to reproductive organs. As a result, any obstetrical emergencies, including post-partum hemorrhage, have an increased probability of precipitating ischemia to the pituitary gland, and subsequently, infarction [1]. Although clinical manifestations of downstream endocrinological effects are somewhat variable, this precipitated hypopituitarism has been well-documented and associated with the development of secondary adrenal insufficiency.

Previous studies further suggest that a diagnosis of Sheehan syndrome can be delayed up to several years. A high index of suspicion, as well as a thorough history, imaging, and endocrinological workup, can confirm the diagnosis [2]. This may be seen in other endocrinological pathological manifestations that are influenced by this physiological axis. To this point, the pathophysiology underlying Sheehan syndrome causing a secondary adrenal insufficiency, insofar as the treatment plan is concerned, will differ when compared to primary adrenal insufficiency. Based on a secondary etiology, treatment would be based on the exogenous replacement of solely glucocorticoids, while a primary etiology would require the additional intervention of exogenous mineralocorticoids. 

In this case study of Sheehan syndrome, we aim to illustrate the importance of evidence-based data-gathering in cases of adrenal crisis, as diagnostic algorithmic approaches in endocrinological diseases, such as Sheehan syndrome, may present similarly to other pathologies of similar etiology. By doing this, future management, therapy, and practices may be guided and focused for overall improvement of care in similar patient demographics.

## Case presentation

A 47-year-old female with a past medical history of hypothyroidism, as well as adrenal insufficiency of previously unknown etiology, presented to the emergency department in adrenal crisis after having a syncopal episode and was subsequently admitted into the intensive care unit for shock. She has had multiple hospitalizations prior for similar presentations for episodes of severe hypotension and fainting. On admission, the patient recounted that she had been prescribed prednisone daily for her adrenal insufficiency, but she forgot to take her doses the last few days. Upon further questioning, the patient had been taking prednisone 40mg daily for approximately 15 years with fludrocortisone intermittently prescribed. She had not been following up with an endocrinologist.

Upon presentation to the emergency department, she was resuscitated with normal saline, hydrocortisone, and fludrocortisone. Additionally, a computed tomography (CT) of her head without contrast was performed, ruling out brain hemorrhage or other acute neurological causes of her syncopal episode. After stabilization in the intensive care unit, she was transferred to the floor for further observation and management.

**Figure 1:**
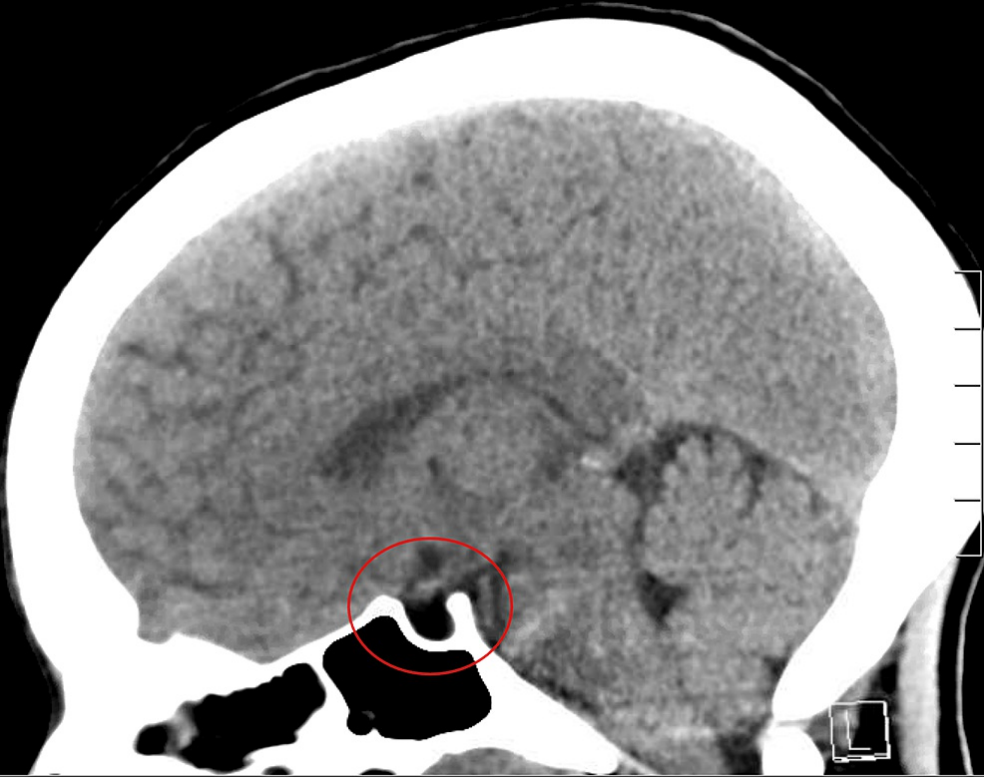
Computed Tomography (CT) without contrast of the head for this patient showing absence of appropriate anatomical structures within the sella turcica

**Figure 2:**
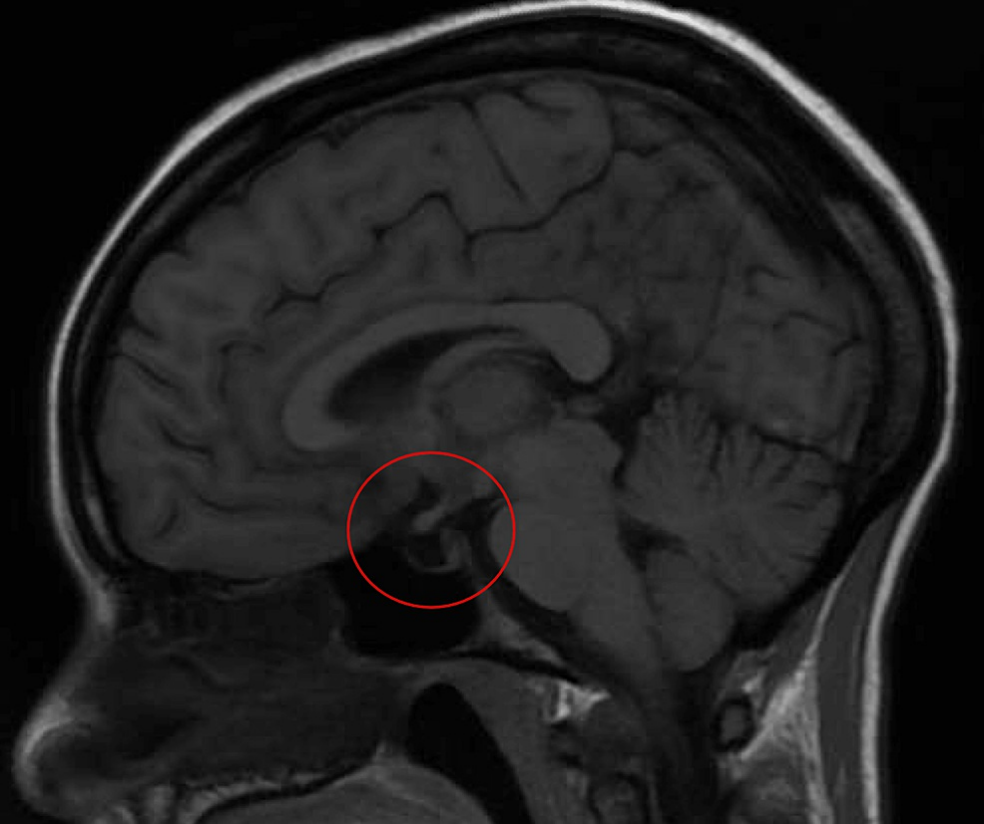
Magnetic resonance imaging (MRI) of the head for this patient confirming the absence of anatomical structures within the sella turcica

On further chart review, the etiology of the patient's adrenal insufficiency was unclear, as there was mention of a possible diagnosis of Addison's disease described by the patient, as well as a partially empty sella from imaging acquired from previous hospitalization, prompting further history gathering from the patient. Further investigation and history revealed three prior pregnancies, each more complicated than the previous one. Further questioning revealed the patient had undergone 'many' blood transfusions during her most recent pregnancy, correlating with initial adrenal insufficiency symptom onset 15 years prior to the current episode. After reviewing the initial CT of the head from the current admission, there was a distinct absence of the pituitary gland in the sella turcica (Figure 1). A subsequent magnetic resonance imaging scan (MRI) was ordered, confirming an empty sella (Figure 2).

With the timeline of the past medical history and confirmatory imaging, the current episode of adrenal crisis was most likely prompted by a lack of medication adherence following a pregnancy-related pituitary infarction that occurred in the patient's most recent pregnancy. A new diagnosis of Sheehan syndrome resulted in impacting the management of the patient's regimen in that she would no longer require fludrocortisone due to the nature of the secondary adrenal insufficiency. The patient was discharged on a 20mg daily prednisone regimen, her fludrocortisone was discontinued, and outpatient follow-up with an endocrinologist was highly recommended.

## Discussion

Several learning points stand out for this particular case when comparing and contrasting the pre-existing literature and understanding of Sheehan syndrome: (1) typical manifestations of Sheehan syndrome have subtle symptoms usually related to thyroid pathology, (2) primary vs. secondary etiology of adrenal insufficiency in Sheehan syndrome has a medical impact on the steroid regimen that should be used to manage these patients, and (3) proper explanation for medical management based on the diagnosis of Sheehan syndrome is imperative not only for the patient, but for future provider use of evidence-based and personalized medicine.

First, although Sheehan syndrome does have a sound physiological background, with symptomatic manifestations that aid in the work-up and thereafter diagnosis, there have been reported cases of delayed diagnosis of Sheehan syndrome. Many times, in prior cases, symptoms have been minor, such as hypoglycemia, fatigue, amenorrhea, myalgia, or episodic vomiting, presenting in an insidious manner [1, 2, 3]. Interestingly, this patient was placed on a long-term multiple steroid regimen prior to admission. What makes this case peculiar is that she was never explained the reason for the steroid regimen, other than she was adrenally insufficient. Understanding the physiological pathways for endogenous steroid production and release, her steroid regimen should have been written based on hypothalamo-pituitary axis dysfunction, not due to a primary cause such as prolonged adrenal ischemia. Other cases have noted that the manifestation of these symptoms is usually from hypothyroid-related issues, such as hypotension, fatigue, and weight changes [1]. This patient's thyroid stimulating hormone (TSH) was 4.42, within normal limits, but she also had a prior history of hypothyroidism. Pathways of thyroid pathology involving steroid production and release, although physiologically possible, such as adrenally related issues, are less reported in cases [2]. 

**Figure 3:**
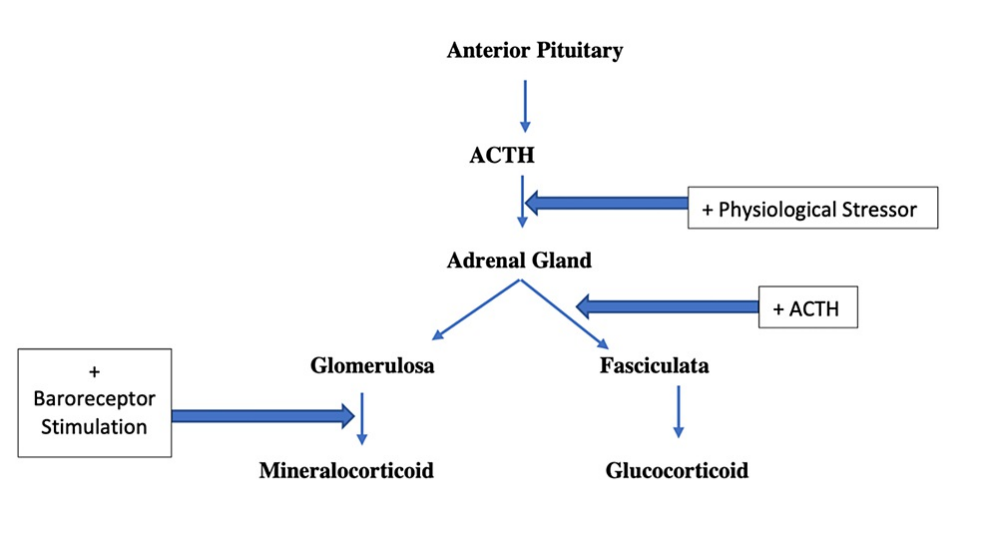
Feedback loop outline for adrenal gland stimulation of steroid release ACTH - adrenocorticotropic hormone Figure generated by and original to the authors.

Second, misdiagnosis of the underlying causes of adrenal crisis, more specifically, primary versus secondary etiology, can lead to inappropriate and ineffective additional steroid regiments. This case demonstrates a secondary etiology of adrenal insufficiency, where the steroid regimen utilized glucocorticoid and mineralocorticoid supplementation, but by understanding the pathophysiology, the mineralocorticoid supplementation was an additional and unnecessary course since physiological mineralocorticoid secretion is primarily influenced by adequate renal perfusion (i.e., renin-angiotensin pathway) and not directly associated with primary adrenal insufficiency (Figure 3). Although steroid regimens mitigate adrenal crisis in Sheehan syndrome, it has also been noted that partial or complete loss of all pituitary hormones and their target organs have been implicated in the pathophysiology of Sheehan syndrome [4]. 

**Figure 4:**
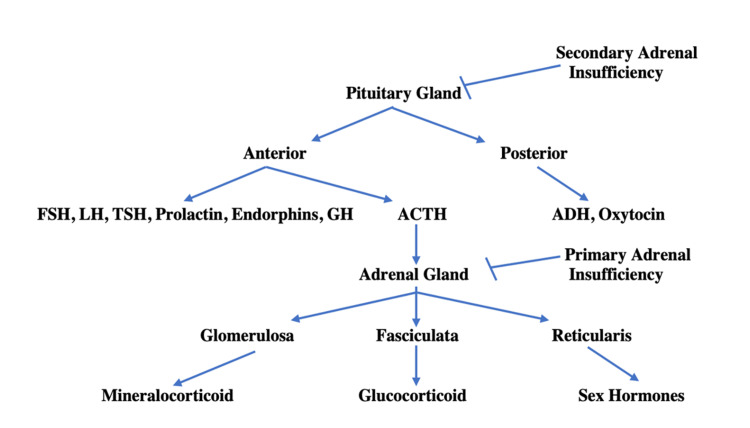
Mechanistic physiological outline of secondary adrenal insufficiency FSH - follicular stimulating hormone, LH - luteinizing hormone, GH - growth hormone, ACTH - adrenocorticotropic hormone, ADH - antidiuretic hormone Figure generated by and original to the authors

The original treatment plan of supplementation was with a mineralocorticoid and a glucocorticoid steroid regimen, which was based on the presumption that the patient's presentation of adrenal insufficiency was based on a primary adrenal etiology. By confirming that this patient was indeed suffering from secondary adrenal insufficiency, discontinuation of mineralocorticoid administration while continuing the glucocorticoid was the appropriate decision based on the physiological understanding of the feedback loops surrounding the stimulation of the associated adrenal gland layers by the pituitary gland (Figure 3). That is, being that physiological mineralocorticoid secretion is mainly stimulated by volume status and adequate renal perfusion pressures, coupled with the secondary adrenal insufficiency etiology itself (i.e., a problem external to the adrenal gland), healthy adrenal glands of this patient could still make mineralocorticoid [5]. The issue that arose in the initial clinical manifestation of the adrenal insufficiency was thus most likely influenced by inadequate neuronal stimulation of the fasciculata layer of the adrenal gland from the pituitary gland, and therefore, the patient would benefit from glucocorticoid supplementation to prevent future adrenal crises (Figure 4).

Finally, unlike in typical scenarios of post-partum complications, possible ongoing endocrine-based manifestations are typically followed up on either with the patient's gynecologist or primary care physician. This case is unusual in that the patient was placed on a long-term multi-steroid regimen without understanding the reasoning. We, the authors, cannot speculate as to why the patient was ill-informed about the medications she was taking or if she was ever explained the ramifications of post-partum hemorrhage from her prior pregnancy so many years ago. Regardless of the matter, it is unclear as to whether she had regularly been seen by a primary care physician or gynecologist, which may perhaps have implications in healthcare accessibility and literacy, but that falls outside the scope of this report on the physiological diagnosis of Sheehan syndrome. These points are important to address in the complete management of a patient's medical condition, as healthcare policy, accessibility, and literacy have a significant impact on the overall long-term prognosis of patients being managed for chronic illnesses.

## Conclusions

We again seek to emphasize the importance of a diagnostic algorithm in approaching adrenal insufficiency, as there are many etiologies that present similarly. By ruling out certain causes with a proficient chart review of previous hospitalizations, diagnosis, and imaging, in addition to targeted follow-up on the history of present illness, an appropriate diagnosis may be made, which will ultimately result in effective and specific therapy and treatment for the patient. We also aim to emphasize the importance of patient education in the setting of new diagnoses and medical management thereafter. This case illustrates the chronicity and severity of Sheehan syndrome management and the sequelae and the importance of appropriate and consistent therapeutic dosing based on the pathophysiology. 
